# The Visual System in Alzheimer’s Disease: A Multilevel Review of Pathology, Monitoring, and Intervention

**DOI:** 10.3390/vision10030050

**Published:** 2026-08-05

**Authors:** Yiman Liu, Junyi Lu, Zile Zhang, Dezhong Yao, Yang Xia, Ke Chen

**Affiliations:** Department of Neurosurgery, Sichuan Academy of Medical Sciences and Sichuan Provincial People’s Hospital, School of Medicine UESTC, University of Electronic Science and Technology of China, No. 2006, Xiyuan Ave., West Hi-Tech Zone, Chengdu 611731, China

**Keywords:** Alzheimer’s disease, visual system, retina, amyloid-β, tau, electroretinography, non-pharmacological intervention

## Abstract

Alzheimer’s disease (AD) extends beyond the brain to the visual system, offering a promising window for pathology, monitoring, and intervention. This review synthesizes evidence on AD-related visual impairments across molecular, cellular, circuit, and cortical levels. We examine the eye–brain pathological relationship as a working framework, noting experimental evidence for brain-to-eye amyloid-β (Aβ) transport in mouse models and associations between retinal and cerebral pathology in humans, while emphasizing that bidirectional pathological transport has not been established. Structural and functional changes in the retina, optic nerve, and visual cortex are reviewed, alongside white matter damage and posterior cortical atrophy patterns. We evaluate emerging multimodal tools (OCTA, ERG, and hyperspectral imaging) that shift diagnosis toward an integrated “structure–vessel–function” assessment. We critically examine non-pharmacological interventions, including 40 Hz gamma stimulation and photobiomodulation, discussing their mechanisms, translational challenges, and the dissociation between structural and cognitive outcomes. We propose the visual system as a candidate pathological window, a quantitative monitoring platform, and an investigational intervention entry point in Alzheimer’s disease. However, clinical translation of these applications requires standardized acquisition protocols, prospective longitudinal validation, and robust mechanistic evidence. To advance this agenda, we identify three priorities: multimodal data integration, closed-loop neuromodulation strategies, and methodologically rigorous validation frameworks.

## 1. Introduction

Alzheimer’s disease (AD) is a neurodegenerative disorder characterized by progressive cognitive impairment and behavioral deficits [1] and is the most common cause of dementia, affecting over 50 million people worldwide [2]. Its histopathological hallmarks are extracellular deposition of amyloid-β (Aβ) plaques and intraneuronal neurofibrillary tangles composed of hyperphosphorylated tau protein [3]. These pathological changes lead to widespread neuronal death, synaptic loss, and brain atrophy [4]. Visual impairment is strongly associated with AD and has been recognized as an important window for disease progression, pathological reflection, prognosis prediction, and potential intervention [5]. Older adults with visual impairment have a 35% higher risk of cognitive decline than those without [6]. As an extension of the central nervous system, the retina and its vascular network exhibit biomarker deposition and inflammatory responses similar to those seen in the brain [7]. Previous studies have indicated that structural retinal changes (e.g., reduced retinal nerve fiber layer [RNFL] thickness), loss of retinal ganglion cells (RGCs), disruption of white matter fiber tract integrity in the visual pathway, and reduced volume and functional metabolism of the lateral geniculate nucleus (LGN) and primary visual cortex (V1) may represent potential mechanisms underlying visual dysfunction [8,9]. This narrative review integrates current evidence on AD-related visual impairment across molecular, cellular, circuit, cortical, and clinical levels. It also critically examines emerging monitoring and intervention approaches and identifies major evidence gaps relevant to future research and clinical translation.

## 2. Materials and Methods

This structured narrative review provides a multilevel synthesis of visual system involvement in Alzheimer’s disease, encompassing molecular, cellular, circuit, cortical, and electrophysiological alterations, together with their translational relevance to disease monitoring and intervention. The objective was not to exhaustively identify or quantitatively pool all available studies, but to integrate representative and directly relevant evidence, clarify relationships across levels of the visual system, and identify current knowledge gaps.

### 2.1. Search Strategy and Databases

A targeted literature search was conducted on 15 March 2026 in PubMed/MEDLINE and Web of Science. Database retrieval was restricted to publications from 1 January 2023 to 15 March 2026; earlier foundational studies were subsequently identified through focused supplementary searches and reference-list screening to establish necessary mechanistic or methodological context. Online-first articles that were accessible by the search date were eligible regardless of their later issue assignment. Only English-language publications were considered. Reference lists of relevant primary studies and reviews were also examined to identify additional sources.

Complete database-specific search strategies are provided in Supplementary Table S1. The prespecified database search cutoff remained 15 March 2026. For the final literature update, the same database-specific search strategies were rerun on 28 July 2026 with the upper date limit advanced to that date; three eligible post-cutoff studies were incorporated during revision.

### 2.2. Search Terms and Structural Framework

Framework searches combined the core terms “Alzheimer’s disease”, “visual system”, “visual impairment”, “retina”, “optic nerve”, “visual pathway”, “visual cortex”, and “eye–brain axis”. Targeted searches were then conducted for the principal domains of the review.

Pathology-related terms included “Aβ”, “tau”, “glymphatic system”, “retinal ganglion cells”, “intrinsically photosensitive retinal ganglion cells” (ipRGCs), “Müller cells”, “microglia”, “synaptic loss”, “white matter”, and “cortical atrophy”. Monitoring-related terms included “optical coherence tomography” (OCT), “OCT angiography” (OCTA), “electroretinography” (ERG), “photopic negative response” (PhNR), “visual evoked potentials” (VEP), “event-related potentials” (ERP), and “hyperspectral imaging”. Intervention-related terms included “40 Hz stimulation”, “gamma entrainment”, “photobiomodulation” (PBM), “transcranial alternating current stimulation” (tACS), and “non-pharmacological intervention”. These thematic terms guided focused supplementary searches and reference-list screening and were not combined as a single additional database query.

### 2.3. Eligibility Criteria and Literature Selection

Eligible sources included original human, animal, and in vitro studies addressing AD-related alterations of the retina, optic nerve, visual pathways, or visual cortex; clinical studies evaluating visual-system biomarkers or interventions; and relevant reviews used to contextualize established mechanisms and emerging findings. Conference abstracts, editorials, non-peer-reviewed preprints, duplicate publications, and studies focused exclusively on primary ocular diseases without a clear relationship to AD pathology were excluded.

**Figure 1:**
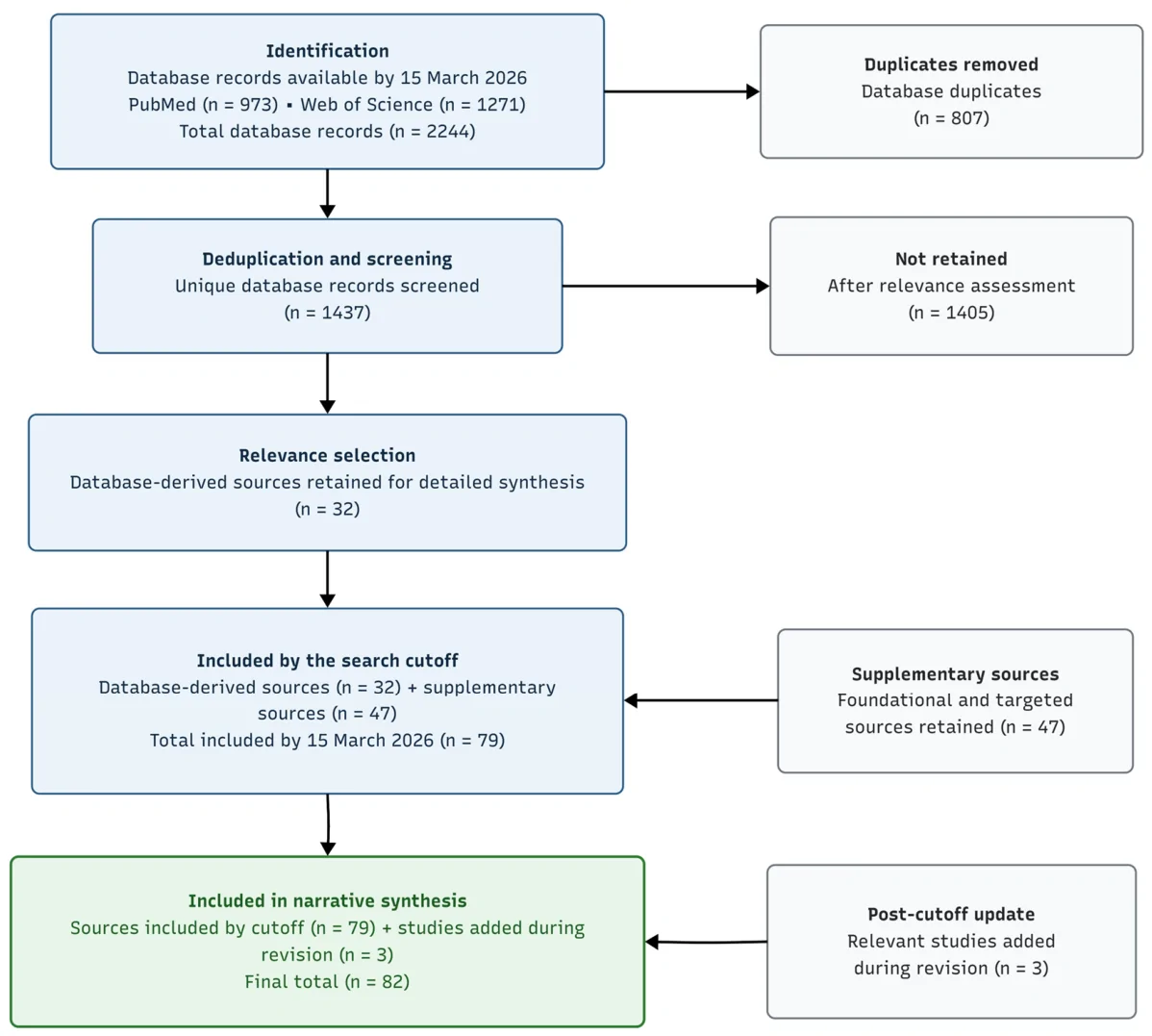
Simplified schematic of the literature identification and selection process for this structured narrative review. The core database search identified 2244 records available in PubMed/MEDLINE and Web of Science by 15 March 2026. After removal of 807 duplicate records, 1437 unique database records underwent relevance assessment, of which 32 were retained for detailed synthesis. An additional 47 foundational and directly relevant sources were included through targeted searches and reference-list screening. Three relevant studies published after the prespecified cutoff were added during revision, resulting in a final total of 82 sources included in the narrative synthesis.

Potentially relevant records were assessed collaboratively by the authors according to the stated thematic eligibility criteria. Differences in judgment regarding inclusion were resolved through discussion among the authors. A simplified schematic of the literature identification and selection process is presented in Figure 1.

### 2.4. Evidence Integration

Information on study design, study population or experimental model, visual-system level, principal findings, and relevant limitations was synthesized qualitatively. Evidence was organized according to two overarching domains: (1) neuropathological manifestations across molecular, cellular, circuit, cortical, and electrophysiological levels; and (2) translational significance involving potential propagation pathways, monitoring approaches, and non-pharmacological interventions. Because this was a narrative review, no formal risk-of-bias assessment or quantitative meta-analysis was performed.

## 3. Neuropathological Manifestations of the Visual System

### 3.1. Molecular-Level Pathology: Aβ, Tau, and the Glymphatic Connection

Under physiological conditions, cerebrospinal fluid–interstitial fluid exchange pathways collectively known as the glymphatic system contribute to the clearance of neurotoxic molecules such as Aβ from the brain. In AD, this clearance is impaired, leading to pathological accumulation. Importantly, this process may extend to the eye. Cao et al. found that increased ocular Aβ accumulation was not accompanied by increased retinal APP or PS1 expression, suggesting that it may not be explained solely by enhanced local retinal production. In mouse tracer experiments, administration into the cisterna magna showed that brain-derived Aβ could travel through the optic nerve subarachnoid space and sheath and enter the retina, accumulating in perivascular spaces (PVS) [10]. These findings provide direct experimental evidence in mice for a brain-to-eye Aβ transport pathway and suggest that this pathway may contribute to AD-related retinal degeneration. Postmortem studies provide additional support: retinal Aβ oligomers are present in patients with mild cognitive impairment (MCI) and AD, with Aβ42 showing significant associations with retinal atrophy and S100β+ macroglial pathology; atrophy severity also parallels neuropathological staging [11,12].

Tau pathology also exhibits a staged expression pattern in the retina. Plasma levels of phosphorylated tau isoforms p-tau181 and p-tau217 are significantly elevated in AD patients and correlate with retinal microvascular damage; p-tau181 shows a particularly strong association and serves as an early marker of Aβ-driven pathology [13]. Cross-sectional analysis of retinal sections from human MCI donors revealed that MC-1+ area percentages overlap considerably between AD and cognitively normal elderly individuals. However, in MCI due to AD, early stages show elevated p-tau pretangle morphology, mid-stages show increased mature tau tangles, and late stages show increased PHF-tau (paired helical filament tau) [14]. These findings suggest that retinal tau pathology may follow a staged evolution similar to that observed in the brain, making it a potentially valuable discriminative marker (summarized in Table 1).

Experimental studies further suggest that glymphatic-like processes may extend to the eye. Findings on aquaporin-4 (AQP4) polarity, Müller cell reactivity, and pericyte integrity indicate biologically linked ocular and cerebral pathology in mouse models, but do not demonstrate bidirectional pathological transport. Chen et al. used APP/PS1: Pdgfr-β+/− mice to show that pericyte loss reduces laminin-211 secretion, disrupting AQP4 polarization on Müller cell endfeet. This disruption severely impairs ocular glymphatic clearance, leading to significant Aβ and p-tau accumulation in the retina, microvascular rarefaction, increased acellular capillaries, and pan-retinal thinning [15]. In APP/PS1 mice, clear RGC synaptic dysfunction (reduced PSD95/VGLUT1 co-localization) and Müller cell activation, indicated by glial fibrillary acidic protein (GFAP) upregulation and loss of AQP4 polarity, preceded plaque-like retinal Aβ deposition, while glutamine synthetase (GS) activity was compensated rather than decreased at early stages [16]. These results suggest that soluble Aβ oligomers may be more pathogenic than plaques in early disease and that glial reactivity may precede and potentially drive neuronal damage. Together, studies of brain-derived Aβ transport, AQP4-dependent clearance, pericyte dysfunction, and glial responses indicate that early retinal pathology involves linked abnormalities in fluid exchange, glial homeostasis, and synaptic function.

### 3.2. Cellular Selective Vulnerability: RGCs, ipRGCs, and Glial Responses

The retina and the central nervous system (CNS) share a common embryonic origin from the neural tube, a relationship reflected in RGCs, whose axons traverse the optic nerve to directly connect the retina to the brain [17]. Among RGC subtypes, ipRGCs are among the earliest neurons affected in AD. In a comprehensive longitudinal study of 3xTg-AD mice aged 4–16 months, Recio et al. observed early dendritic beading and reduced pupillary light reflex under scotopic conditions, followed later by decreased ipRGC numbers and diminished pupillary light reflex under photopic conditions. These structural and functional changes preceded cognitive decline in behavioral tests, supporting the hypothesis that ipRGC dysfunction occurs very early in AD pathogenesis [18]. These experiments included both male and female mice, but did not report sex-stratified analyses. Future studies should address this limitation given known sex differences in AD epidemiology.

In human postmortem samples, Davis et al. found that RGC counts declined in MCI and AD patients, and that the number of RBPMS-positive RGCs containing pS396-tau or oligomeric tau increased significantly. This tau accumulation correlated with RGC degeneration, and RGC loss correlated with cognitive scores (Clinical Dementia Rating and Mini-Mental State Examination), suggesting a direct link between retinal neurodegeneration and AD-related cognitive function [19]. In vivo ERG studies detected RGC functional loss even in early-stage AD patients, with PhNR providing the most stable and reliable metric. Postmortem tissues from severe AD cases also confirmed marked retinal atrophy, particularly in the inner RGC layers [20].

Beyond RGCs, other cell types also exhibit AD-related pathology. Using GFAP and GS immunolabeling, Xu et al. demonstrated that macroglia (astrocytes and Müller cells) showed reduced immunoreactivity in AD eyes compared with controls, whereas microglial immunoreactivity was enhanced. This pattern indicates impaired Müller cell function and activated microgliosis in the AD retina, suggesting that glial dysfunction plays a critical role alongside Aβ deposition [21]. As reviewed by Leng and Edison, microglia can be modulated at different stages of the AD trajectory and may therefore represent intervention targets [22]. The observed cellular selectivity warrants investigation of why some retinal cell types are more vulnerable than others, which may inform cell-targeted therapeutic strategies.

### 3.3. Circuit-Level Dysfunction: Synaptic Loss and Projection Imbalance

Synaptic loss and dysfunction are hallmark features of AD pathophysiology and are directly linked to Aβ deposition [23]. In a landmark study, Hong et al. demonstrated that when resident microglia in the adult CNS are exposed to oligomeric Aβ (oAβ), they engulf synapses via complement-dependent mechanisms, identifying microglia as a cellular mediator of synaptic loss [24]. ERG recordings from 8-month-old 3xTg-AD mice showed significantly reduced scotopic b-wave amplitudes compared with wild-type controls, indicating reduced neuronal activity or connectivity in the retinal circuit. While the postsynaptic marker PSD-95 did not change significantly, the presynaptic marker synaptophysin was significantly reduced in the inner plexiform layer (IPL) of these mice. This decreased presynaptic protein expression suggests impaired connectivity between retinal neurons, providing a structural basis for information processing deficits [25]. Beyond local synaptic changes, AD can affect higher-order visual projection circuits. Nam et al. extended this circuit-level analysis to the eye-to-brain pathway using 5xFAD mice overexpressing Aβ and retrograde tracing with Alexa Fluor 488-conjugated cholera toxin subunit B (CTβ) injected into the nasal retina. They demonstrated significant impairment of retinal projections to the suprachiasmatic nucleus (SCN), dorsal lateral geniculate nucleus (dLGN), intergeniculate leaflet (IGL), and ventral lateral geniculate nucleus (vLGN) in early AD. In late AD, retinal connections to the dLGN and superior colliculus (SC) were additionally disrupted. These findings reveal mesoscale visual circuit disorganization that progresses with disease stage, affecting both circadian and visual processing pathways [26].

### 3.4. Cortical Atrophy and White Matter Tract Damage

The aforementioned studies focus predominantly on retinal pathology; we now extend our analysis to the visual pathway proper. Using APP/PS1 mice in a spatiotemporal analysis, Chen et al. [27] reported that pathological alterations in V1 emerged earlier than those in the LGN. Aβ deposition and neuroinflammation in V1 appeared synchronously with hippocampal changes and earlier than spatial working memory deficits; at 9 months, V1 showed selective neuronal loss (with relative preservation of parvalbumin-positive interneurons), but cortical thickness remained unchanged, suggesting compensatory mechanisms. This study highlights the central role of V1 in visual pathway damage and provides a strong rationale for early diagnostic strategies based on V1 function, such as VEP [27].

Posterior cortical atrophy (PCA) typically presents with progressive decline in higher-order visual processing and has been shown to be highly specifically associated with underlying AD pathology [28]. Voxel-based morphometry (VBM) remains one of the most widely used methods to study whole-brain atrophy in AD [29,30]. In 2026, Licciardo et al. provided the most comprehensive meta-analysis of VBM data in PCA to date [31]. Their results showed consistent gray matter (GM) loss in occipital, parietal, precuneus, and ventral temporal regions, summarized as a characteristic bilateral GM atrophy pattern with the most significant involvement in the right lateral occipital cortex (extending to the inferior parietal lobule and ventral occipitotemporal region), the left superior occipital gyrus, and the bilateral precuneus. Compared with the earlier meta-analysis by Alves et al. (2013) [32], this new study indicates more pronounced right-lateralized damage, particularly in occipital and temporal cortices, and involves broader dorsal and ventral visual pathways. These affected regions encompass fiber tracts critical for visuospatial integration, dorsal/ventral stream functions, and visuomotor coordination, providing key evidence for understanding AD-related visual deficits [33].

In addition to cortical atrophy, diffusion tensor imaging (DTI) studies have provided convergent evidence. Nishioka et al. were among the first to use DTI to confirm visual pathway damage in MCI and AD patients, showing that the corpus callosum is affected during disease progression [34]. Building on this, Risacher et al. integrated neuroimaging findings to reveal Aβ plaques and GM atrophy in AD patients [35]; Christopher Nishioka et al. further proposed a mechanism by which visual white matter tract damage contributes to visual impairment. They confirmed that MCI patients show increased trace diffusion and radial diffusivity and decreased fractional anisotropy (FA) in the optic nerve and genu of the corpus callosum, indicating that white matter damage extends to the visual pathway and that disrupted connectivity directly affects neural networks [34]. Importantly, conventional DTI has limited capacity to resolve crossing fiber regions; future studies should incorporate higher-order diffusion models (e.g., constrained spherical deconvolution, neurite orientation dispersion and density imaging) and structural connectomics to characterize visual-pathway white matter damage more accurately. Chen et al., from a multilevel white matter hierarchy perspective, further demonstrated that MCI patients exhibit extensive white matter lesions at the tract level and that damage is closely related to multidomain cognitive impairment, including visuospatial function, with the brain’s structural connectome playing a key mediating role [36]. This supports the theory of white matter disconnection in early cognitive decline and provides mechanistic evidence linking prodromal white matter degeneration to cognitive impairment.

### 3.5. Neuroelectrophysiological Functional Manifestations

Neuroelectrophysiological indices provide quantitative functional tools for assessing visual impairment in AD. ERPs are non-invasive measures with millisecond precision and are ideally suited for studying perceptual and cognitive processes in both healthy individuals and patients with AD [37,38]. For example, ERP components such as N200 and P300 are highly correlated with disease severity [39,40], while P200 is associated with sensory and attentional processes [41,42]. Although these components have been widely used to evaluate AD-related cognitive changes, those more directly linked to visual impairment include VEP and face-processing components such as P100, N170, and VPP.

Pattern-reversal VEP is the most clinically established visual electrophysiological test. It typically shows prolonged P100 latency and reduced amplitude in AD patients; these findings indicate non-specific abnormalities of visual-pathway conduction and/or cortical processing rather than demyelination per se. These changes correlate with retinal nerve fiber layer thinning and cognitive scores, making VEP a valuable adjunctive tool. As the disease progresses, AD patients may develop prosopagnosia because of both memory impairment [43] and deficits in higher-order visual processing. N170 is a classic visual ERP component reflecting face processing, peaking at 140–200 ms after face presentation [44,45], and mainly represents the perception of spatial configural features and discrimination of individual facial identity [46]. Lazarou et al. found that compared with healthy elderly individuals, AD patients showed reduced N170 amplitude during facial visual stimulation, suggesting that N170 amplitude may be affected by pathological cognitive decline when processing negative emotional faces [47]. Fide et al. designed a passive viewing task of emotional facial expressions and found that N170 amplitude was significantly reduced in AD patients, associated with impaired perceptual processing [48]. Additionally, Saavedra et al. found that AD patients had enhanced anterior VPP amplitude, possibly related to prefrontal dysfunction, while posterior N170 amplitude was reduced, indicating that N170 and VPP may be jointly affected by pathological cognitive aging [49]. Fide et al. also found that P100 amplitude and latency were significantly increased in AD patients compared with controls, possibly reflecting reduced amygdala volume and disruption of the visual system [48]. Marin et al. reported that P200 parameter changes achieved an 84% detection rate for memory-impaired patients and showed high specificity and sensitivity in predicting Aβ PET status, and that differences in P200 amplitude and latency between PET-positive and PET-negative groups may indicate compensatory mechanisms and sensory gating deficits related to underlying neurodegeneration [50]. The visual mismatch negativity (vMMN) has been proposed as a candidate electrophysiological index of early visual perceptual anomalies, but direct evidence specifically in AD cohorts remains sparse and requires systematic validation in future longitudinal studies. These findings support further evaluation of VEP and face-sensitive ERPs as candidate markers for identifying individuals at high risk of AD.

## 4. Translational Significance of the Visual System in AD

### 4.1. Eye–Brain Relationship: Co-Existing Pathology and Experimental Brain-to-Eye Transport

Compared with AD patients without visual impairment, those with visual impairment exhibit more severe deficits in global cognition, memory, language, attention, and executive function [51]. Evidence across animal and human studies supports investigation of an eye–brain pathological relationship, but its components should be distinguished: brain-to-eye Aβ transport has been demonstrated in experimental mouse models, whereas human studies currently show associations between retinal and cerebral pathology rather than a confirmed transport pathway.

For a long time, retinal Aβ deposition was thought to originate mainly from local amyloid precursor protein processing, and retinal Aβ plaques were considered necessary for retinal damage [52]. However, Cao et al., using intracisternal administration of tracers in 5xFAD mice, directly demonstrated that brain-derived Aβ can be transported to the retina via the peri-optic nerve glymphatic-like pathway and accumulate in perivascular spaces and Müller cell endfeet [10]. This finding challenges and complements the traditional view that retinal Aβ is primarily produced locally. Importantly, AQP4 deficiency slowed brain-to-eye Aβ transport in the short term but reduced the long-term clearance of ocular Aβ through the perivenous space–optic nerve meningeal lymphatic pathway, thereby increasing Aβ retention and aggravating retinal damage [10]. These findings indicate that AQP4 contributes both to brain-to-eye influx and to subsequent ocular clearance, with its deficiency resulting in both slower input and slower output.

However, several questions remain unresolved. First, does transport from the retina to the brain also occur, and if so, does it contribute to central pathology? Current evidence is insufficient to confirm a bidirectional pathway, and future studies using dual-tracer techniques or longitudinal sampling are needed. Second, what is the temporal sequence and dose–response relationship between brain Aβ burden and retinal deposition? Cross-sectional data cannot establish causality or predict whether retinal changes precede or follow cortical changes in individual patients. Third, is this propagation specific to Aβ, or does it extend to tau and other misfolded proteins? These open questions define a clear agenda for future mechanistic research. Thus, current evidence supports brain-to-eye Aβ transport in experimental mouse models and an association between retinal and cerebral pathology in humans, while the existence of a bidirectional pathological continuum in human AD remains unproven. The glymphatic system is therefore best regarded as a plausible mechanistic contributor that requires further validation.

### 4.2. Visual System as a Disease Monitoring Window

Based on reported associations between ocular and cerebral pathology, the retina has been investigated as a non-invasive window for AD monitoring [53]. Traditional OCT metrics include peripapillary retinal nerve fiber layer (pRNFL) thickness and ganglion cell–inner plexiform layer (GC-IPL) thickness, which have been validated as biomarkers in some studies [54]. However, challenging this single-metric reliance, Banihashemi et al., after pooling multiple studies, questioned its diagnostic value: the area under the curve (AUC) for pRNFL thickness distinguishing AD from controls was only 0.57–0.70, and for GC-IPL distinguishing MCI from controls, the AUC was as low as 0.55–0.72. Moreover, thickness differences between groups often fell below OCT resolution (5–7 μm). After adjusting for confounders such as head circumference and age, the standardized regression coefficient between retinal thickness and hippocampal volume was only −0.03 to −0.06, suggesting limited clinical utility [55]. We interpret these findings as strong evidence that conventional thickness metrics should not be used as standalone screening tools but may retain value as adjunctive measures within multimodal panels.

Wang et al. addressed this limitation by developing a gray-level co-occurrence matrix algorithm to extract retinal OCT intensity spatial correlation features (OCT-ISCF), including angular second moment, correlation, and homogeneity. In a test of 82 subjects, they showed that OCT-ISCF enhanced the association with AD pathology and detection performance compared with single thickness metrics [56]. In contrast, choroidal thickness can differ by up to 65 μm between AD and controls; OCTA-measured vessel density and foveal avascular zone (FAZ) show reproducible changes at the MCI stage and correlate with cognitive scores [57].

Electrophysiological measures, optical imaging, and fluid biomarkers have also shown potential as complementary monitoring approaches. AD is associated with generally reduced full-field ERG amplitudes. Among all electrophysiological indices, b-wave amplitude showed the highest discriminative ability for distinguishing AD from healthy controls (AUC = 0.956), followed by PhNR amplitude (AUC = 0.930) and a-wave amplitude (AUC = 0.904) [58]. The PhNR amplitude was significantly reduced even in asymptomatic individuals with Aβ-PET positivity, differentiating preclinical AD from controls with AUCs of 0.84–0.90. Hyperspectral imaging (HSI), without requiring exogenous fluorescent probes, captures wavelength-dependent changes in retinal reflectance and has shown potential for distinguishing individuals with high versus low cerebral Aβ burden, with an AUC of up to 0.89 [59]; combining HSI with OCT further improves AD detection accuracy [60]. Additionally, decreased tear Aβ1-42 and elevated t-tau showed an AUC of 0.91 for distinguishing MCI/AD from controls and correlated with cortical thickness [61].

In 2026, Salajková et al. evaluated a custom tri-spectral retinal imaging module integrated with an existing fundus imaging system. In an initial case–control analysis of 38 patients with biomarker-confirmed mild AD and 28 age-matched controls, the blue-to-green retinal reflectance ratio differed significantly between groups (*p* < 0.001), but the standalone spectral measure achieved an AUC of 0.74 (95% CI: 0.61–0.86). In an expanded dataset comprising 79 participants and 138 eyes, an XGBoost classifier combining retinal spectral features with age, sex, and previous ocular interventions achieved an AUC of 0.83 (95% CI: 0.75–0.91), with an accuracy of 75.4%, sensitivity of 82.4%, and specificity of 66.0% under leave-one-out cross-validation. In the held-out test set, the combined model achieved an AUC of 0.91 (95% CI: 0.78–1.00), with an accuracy of 85.0%, sensitivity of 84.6%, and specificity of 85.7%. Thus, the reported AUC of 0.91 reflects the performance of the combined spectral-clinical-demographic model rather than tri-spectral retinal imaging alone. The limited size of the held-out test set and the wide confidence interval indicate that larger prospective cohorts and external validation are still required [62].

However, the specificity and generalizability of these candidate biomarkers remain limited. OCT- and OCTA-derived measurements may be influenced by age, ocular and systemic comorbidities, image quality, device platforms, scan protocols, and segmentation procedures. Electrophysiological measures such as ERG and PhNR may also be affected by coexisting retinal disease and acquisition conditions, whereas spectral imaging can be influenced by media opacity, illumination, pigmentation, calibration, and image-registration procedures. These visual-system measures should therefore be interpreted as adjunctive components of multimodal assessment rather than as AD-specific standalone diagnostic tests. Future studies should exclude or stratify relevant ocular and systemic comorbidities, harmonize acquisition and analysis protocols, report explicit quality-control criteria, and validate candidate biomarkers longitudinally against established AD biomarkers [55,61,62].

### 4.3. New Intervention Directions Starting from the Visual System

Current pharmacotherapies have made progress in disease-modifying directions such as Aβ clearance [63]. However, a 2026 Cochrane review of 17 randomized controlled trials involving 20,342 participants with MCI or mild dementia due to AD concluded that Aβ-targeting monoclonal antibodies (Aβ-mAbs) produced trivial effects on cognitive function and dementia severity at 18 months and, at best, small effects on functional ability, while increasing the risk of amyloid-related imaging abnormalities [64]. A Japanese research team further noted that in individual cases, no correlation was observed between Aβ reduction and inhibition of clinical symptom progression, and that minimal clinically important differences could not be inferred from Aβ reduction alone [65]. Currently, including cholinesterase inhibitors and NMDA receptor antagonists, no conventional drug therapy can effectively halt the natural course of AD toward progressive disability [66].

This therapeutic gap has prompted increasing interest in non-pharmacological interventions. Critically, visual impairment not only directly affects cognitive function but also indirectly impacts cognition through social isolation and depressive symptoms [67]. Cognitive stimulation, cognitive training, and cognitive rehabilitation may support cognition and daily functioning in people living with dementia [68]. However, these interventions do not directly target AD-related visual pathology and should be distinguished from visual-system-based neuromodulation.

Observed brain-to-eye Aβ transport has prompted investigation of the visual system as a potential route for intervention. Such approaches should be evaluated separately from conventional pharmacotherapy, and their mechanistic and clinical relevance remains to be established.

At the preclinical level, 40 Hz sensory stimulation uses rhythmic visual and/or auditory input to entrain endogenous gamma activity. In 5xFAD mice, Murdock et al. found that multisensory gamma stimulation increased cerebrospinal fluid influx and interstitial fluid efflux, enhanced arterial pulsation, and promoted AQP4-dependent glymphatic clearance of Aβ through a pathway involving vasoactive intestinal peptide-expressing interneurons [69]. These findings provide mechanistic evidence from an animal model, rather than evidence of clinical efficacy in humans.

At the human clinical level, the evidence is more limited. A meta-analysis of 11 studies involving 341 participants found that gamma-frequency auditory and visual stimulation was associated with changes in neuroimaging or cerebrospinal fluid biomarker outcomes, with a standardized mean difference (SMD) of 1.74 (95% CI: 0.31–3.18; *p* = 0.02), but did not significantly improve cognitive function (SMD = 0.16, 95% CI: −0.36 to 0.68; *p* = 0.55) or activities of daily living (SMD = 0.53, 95% CI: −1.26 to 2.33; *p* = 0.56). Substantial heterogeneity, particularly for structural and functional outcomes, further limits the certainty and generalizability of these findings [70].

PBM is mechanistically distinct from 40 Hz sensory entrainment. PBM applies red or near-infrared light, commonly at wavelengths of approximately 810 or 1060–1080 nm, through transcranial or intranasal routes. Its proposed mechanisms include modulation of mitochondrial metabolism, cerebral blood flow, vascular and blood–brain barrier function, and inflammatory responses rather than frequency-specific entrainment of gamma oscillations. Although small clinical studies have reported trends toward cognitive or functional improvement, the available evidence is limited by small samples, heterogeneous wavelengths and doses, variable treatment routes, and a lack of sufficiently powered randomized controlled trials [71]. Accordingly, the effects of PBM on human AD pathology and long-term clinical outcomes remain uncertain. Integration of PBM with OCT or ERG monitoring should currently be regarded as a prospective research framework rather than an established closed-loop therapeutic system.

Additional animal studies further illustrate the protocol dependence of 40 Hz gamma induction. Gamma oscillations at 40 Hz play a fundamental role in neural coordination [72] and support complex cognitive processes such as memory [73]; moreover, their dysfunction is a hallmark of AD pathophysiology [74]. Wang et al. argue that although this concept is theoretically attractive, its therapeutic efficacy depends on experimental precision and rigorous translational validation [75]. Early animal studies showed significant benefits of 40 Hz sensory stimulation. For instance, Iaccarino et al. demonstrated that 40 Hz visual flicker in transgenic AD mice synchronized cortical gamma oscillations, reduced Aβ load, and modulated microglial function [76]. However, subsequent results have been inconsistent. Wilson et al. highlighted that optogenetic activation of basal forebrain parvalbumin neurons at 40 Hz unexpectedly increased Aβ42 plaque deposition, emphasizing that the mode of gamma induction and targeted neural substrates critically influence outcomes [77]. Soula et al. further reported that 40 Hz light stimulation failed to effectively induce gamma oscillations in AD mouse models and had minimal effect on Aβ burden [78]. These apparently conflicting animal findings indicate that pathological effects are protocol- and circuit-dependent and cannot be directly extrapolated to therapeutic efficacy in humans.

Gamma-frequency tACS represents a third, distinct intervention that applies weak alternating electrical currents to modulate cortical oscillations directly, rather than using sensory input or photobiological mechanisms. A systematic review found that gamma-tACS was generally feasible and tolerable, but emphasized that the available dementia studies were small and heterogeneous [79]. In a randomized clinical trial involving 50 patients with prodromal or mild AD, home-based gamma-tACS was well tolerated and was associated with improvements in clinical and neurophysiological outcomes; however, no changes in plasma AD biomarkers were detected [80]. An earlier open-label study involving 15 patients reported increased temporal-lobe perfusion after multisession 40 Hz tACS, with perfusion changes correlating with episodic-memory and gamma-power changes, but the absence of a sham-control group limits causal interpretation [81]. This suggests that in humans, gamma synchronization may primarily involve network-level functional plasticity rather than directly altering molecular pathology. Thus, current human evidence supports brain engagement and possible network-level effects, but does not yet establish modification of Aβ pathology or durable clinical efficacy.

Human evidence for 40 Hz sensory stimulation also remains preliminary. In a Phase 2A pilot study involving 15 patients with mild probable AD dementia, combined visual and auditory stimulation was feasible and well tolerated, but the study was not powered to evaluate clinical efficacy. No significant between-group differences were detected in MMSE, MoCA, ADAS-Cog, or CDR scores, although exploratory changes in selected structural, connectivity, and associative-memory measures were reported [82]. Taken together, 40 Hz sensory stimulation, PBM, and gamma-tACS should be evaluated as separate modalities with distinct mechanisms, stimulation parameters, and outcome measures. Future trials should use adequately powered randomized designs, standardized stimulation protocols, objective cognitive and visual outcomes, and established AD biomarkers while avoiding direct extrapolation from animal pathological changes to human therapeutic efficacy.

## 5. Summary and Perspectives

In summary, AD-related visual impairment is not a single decline in visual function, but rather an integrated manifestation of multi-level changes including molecular pathology deposition, glial homeostasis imbalance, selective RGC vulnerability, retinal microvasculature, visual pathway connectivity, and degeneration of higher-order visual cortical networks. Recent evidence concerning brain-derived Aβ transport, AQP4-related glymphatic dysfunction, and Müller cell reactivity supports a working model of an eye–brain pathological relationship, based primarily on experimental brain-to-eye transport findings and associative human evidence. However, the extent, disease specificity, and clinical significance of these relationships remain incompletely established, particularly in humans.

For disease monitoring, multimodal approaches combining OCT/OCTA, visual electrophysiology, and spectral or molecular measures may be more informative than any single structural metric. Nevertheless, ocular and systemic confounders, device and protocol heterogeneity, and the lack of longitudinal external validation currently limit their diagnostic specificity. Similarly, 40 Hz sensory stimulation, PBM, and tACS remain investigational and should not be interpreted as established treatments for AD-related visual impairment.

In the future, the visual system may simultaneously serve triple roles: a window for pathological observation, a platform for disease monitoring, and an entry point for regulation and intervention. Future research should prioritize standardized acquisition and analysis protocols, prospective longitudinal cohorts linked to established AD biomarkers, explicit control of ocular comorbidities, and adequately powered randomized trials with modality-specific cognitive, visual, and biological outcomes. Within this framework, the visual system may serve as a source of pathological and multimodal monitoring information. Its use as a route for neuromodulation remains investigational.

## Figures and Tables

**Table 1:** Overview of selected studies on molecular, cellular, vascular, and synaptic pathology of the visual system in Alzheimer’s disease.

Study.	Model System	Finding	Mechanistic Implication
Cao et al., 2024 [10]	5xFAD mice	Brain-derived Aβ transports to the retina via optic nerve sheath; deposits in PVS and Müller cell endfeet	In this mouse model, brain-derived Aβ contributes to retinal deposition, challenging a local-production-only model
Koronyo et al., 2023 [12]	Postmortem human eyes/brain (MCI, AD)	Retinal Aβ42 accumulation correlates with brain pathology and cognitive scores	Retinal Aβ42 was associated with cerebral pathology and cognitive measures in this postmortem study
Kwapong et al., 2025 [13]	Cross-sectional clinical study (72 patients with AD and 82 cognitively unimpaired controls)	Retinal microvascular damage (reduced density, branching) correlates with elevated p-tau181/p-tau217	Tau abnormalities contribute to retinal vascular injury; p-tau181 is a stronger correlate
Shi et al., 2024 [14]	Human eye donors	First evidence of hypercitrullinated tau in MCI and AD retinas; increased MC-1+ tau tangles are key in disease progression	Retinal tau conformers follow staged evolution similar to brain tauopathy
Chen et al., 2026 [15]	APP/PS1: Pdgfr-β+/− mice	Pericyte loss → reduced laminin-211 → loss of AQP4 polarization → impaired glymphatic clearance → retinal Aβ/tau accumulation	The pericyte-AQP4 axis is critical for ocular glymphatic function
Zhou et al., 2026 [16]	APP/PS1 mice (pre-plaque stage)	RGC synaptic dysfunction (PSD95/VGLUT1 ↓) and Müller cell activation (GFAP ↑, AQP4 polarity loss) precede plaque deposition	Soluble Aβ oligomers and glial reactivity drive early retinal pathology

Note: → indicates the proposed pathological sequence; ↑ and ↓ indicate increased and decreased expression, respectively.

## Data Availability

No new data were created or analyzed in this study. Data sharing is not applicable to this article.

## Supplementary Materials

The following supporting information can be downloaded at https://www.mdpi.com/article/10.3390/vision10030050/s1, Table S1: Complete database-specific search strategies and the literature update details.

## Abbreviations

The following abbreviations are used in this manuscript:

AD	Alzheimer’s disease
Aβ	amyloid-β
AQP4	Aquaporin-4
AUC	Area under the curve
CNS	Central nervous system
DTI	Diffusion tensor imaging
dLGN	Dorsal lateral geniculate nucleus
ERG	Electroretinography
ERP	Event-related potential
FAZ	Foveal avascular zone
GC-IPL	Ganglion cell–inner plexiform layer
GFAP	Glial fibrillary acidic protein
GM	Gray matter
HSI	Hyperspectral imaging
IGL	Intergeniculate leaflet
ipRGC	Intrinsically photosensitive retinal ganglion cell
LGN	Lateral geniculate nucleus
MCI	Mild cognitive impairment
OCT	Optical coherence tomography
OCTA	Optical coherence tomography angiography
PBM	Photobiomodulation
PCA	Posterior cortical atrophy
PhNR	Photopic negative response
pRNFL	Peripapillary retinal nerve fiber layer
PVS	Perivascular space
RGC	Retinal ganglion cell
RNFL	Retinal nerve fiber layer
SC	Superior colliculus
SCN	Suprachiasmatic nucleus
SMD	Standardized mean difference
tACS	Transcranial alternating current stimulation
V1	Primary visual cortex
VBM	Voxel-based morphometry
VEP	Visual evoked potential
vLGN	Ventral lateral geniculate nucleus
WM	White matter
