# Supplementary material for: The Visual System in Alzheimer’s Disease: A Multilevel Review of Pathology, Monitoring, and Intervention
Source: Vision (Basel). 2026 Aug 5;10(3):50. doi: 10.3390/vision10030050 (PMC13517210; doi:10.3390/vision10030050)
Supplement: Supplementary file 1 [file vision-10-00050-s001.zip › vision-4441148-supplementary.pdf]

Supplementary Material: The Visual System in Alzheimer’s Disease: A Multilevel Review of Pathology, Monitoring, and Intervention

Yiman Liu, Junyi Lu, Zile Zhang, Dezhong Yao, Yang Xia and Ke Chen

Supplementary Table S1. Complete database-specific search strategies and the literature update details.

Database and search details	Exact strategy, date handling, and results
**Database: PubMed/** MEDLINE<br>Initial search cutoff: 15 March 2026<br>Query-stage language filter: None<br>Publication-date range: 1 January 2023 to 15 March 2026<br>Initial records: n = 973	("Alzheimer Disease"[Mesh] OR Alzheimer*[Title/Abstract])<br>AND<br>(<br>"visual system"[Title/Abstract]<br>OR "visual impairment"[Title/Abstract]<br>OR retina*[Title/Abstract]<br>OR "optic nerve"[Title/Abstract]<br>OR "visual pathway"[Title/Abstract]<br>OR "visual pathways"[Title/Abstract]<br>OR "visual cortex"[Title/Abstract]<br>OR "eye-brain axis"[Title/Abstract]<br>OR "optical coherence tomography"[Title/Abstract]<br>OR OCT[Title/Abstract]<br>OR OCTA[Title/Abstract]<br>OR electroretinograph*[Title/Abstract]<br>OR ERG[Title/Abstract]<br>OR "photopic negative response"[Title/Abstract]<br>OR PhNR[Title/Abstract]<br>OR "visual evoked potential"[Title/Abstract]<br>OR "visual evoked potentials"[Title/Abstract]<br>OR "event-related potential"[Title/Abstract]<br>OR "event-related potentials"[Title/Abstract]<br>OR "hyperspectral imaging"[Title/Abstract]<br>OR "40 Hz"[Title/Abstract]<br>OR "40-Hz"[Title/Abstract]<br>OR "gamma entrainment"[Title/Abstract]<br>OR photobiomodulation[Title/Abstract]<br>OR "transcranial alternating current stimulation"[Title/Abstract]<br>OR tACS[Title/Abstract]<br>)<br>AND ("2023/01/01"[dp] : "2026/03/15"[dp])<br><br>Fields and limits: [Mesh] and [Title/Abstract] fields; publication-date range specified with [dp]. The main PubMed search was intentionally restricted to publications dated from 1 January 2023 through 15 March 2026.

**Database and search details**	**Exact strategy, date handling, and results**
**Database: Web of Science Core Collection**<br>Initial search cutoff: 15 March 2026<br>Field: Topic (TS)<br>Query-stage language filter: None<br>Publication-year filter: 2023–2026<br>Initial records: n = 1271	TS=(Alzheimer* AND (<br>"visual system" OR "visual impairment" OR retina* OR "optic nerve"<br>OR "visual pathway" OR "visual pathways" OR "visual cortex"<br>OR "eye-brain axis" OR "optical coherence tomography"<br>OR OCT OR OCTA OR electroretinograph* OR ERG<br>OR "photopic negative response" OR PhNR<br>OR "visual evoked potential" OR "visual evoked potentials"<br>OR "event-related potential" OR "event-related potentials"<br>OR "hyperspectral imaging" OR "40 Hz" OR "40-Hz"<br>OR "gamma entrainment" OR photobiomodulation<br>OR "transcranial alternating current stimulation" OR tACS<br>))<br><br>Date handling: The initial search was restricted to publication years 2023–2026. Because the interface applied year-level filtering, 2026 records were exported and records dated after 15 March 2026 were removed after export. Topic (TS) searches title, abstract, author keywords, and Keywords Plus.
**Final update: 28 July 2026**<br>Approach: Same database-specific concepts and fields; 2023 lower date bound retained and upper cutoff advanced to 28 July 2026<br>Update exports: PubMed n = 1016; Web of Science n = 1404 unique records<br>Eligible post-cutoff studies added: n = 3<br>Final synthesis: n = 82 sources	The searches were rerun on 28 July 2026 using the same search terms and fields while retaining the 2023 lower date bound. For PubMed, the [dp] range was updated to 1 January 2023 through 28 July 2026. For Web of Science, the 2023–2026 publication-year filter was retained and records dated after 28 July 2026 were removed after export. The PubMed export contained 1016 records. The two Web of Science export batches contained 1000 and 405 records; one duplicate UT occurred across the batches, yielding 1404 unique Web of Science records. Newly available records were assessed against the same eligibility criteria. Three eligible studies published after the prespecified cutoff were incorporated during revision. Together with 32 database-derived sources and 47 supplementary sources identified through focused searches and reference-list screening, the final narrative synthesis included 82 sources.

Note: No language filter was included in either database query; non-English publications were excluded during eligibility screening. The main database searches were intentionally limited to publications from 2023 onward. Pre-2023 sources cited in the final manuscript were identified during iterative full-text reading and backward reference tracing of eligible articles, with focused supplementary searches where needed; they were included when they provided relevant original or foundational evidence. Thus, the final set of 82 cited sources was not restricted to 2023–2026. The manuscript's typographic form "amyloid-β" is represented by the searchable terms used in the strings above. Live database counts may change because of retrospective indexing or record updates.
